# Changes in heart failure medications in patients hospitalised and discharged

**DOI:** 10.1186/1471-2296-7-69

**Published:** 2006-11-23

**Authors:** Martin Scherer, Cordula Sobek, Dirk Wetzel, Janka Koschack, Michael M Kochen

**Affiliations:** 1 Department of General Practice, University of Göttingen, Humboldtallee 38, 37073 Göttingen, Germany

## Abstract

**Background:**

To date, evidence-based recommendations help doctors to manage patients with heart failure (HF). However, the implementation of these recommendations in primary care is still problematic as beneficial drugs are infrequently prescribed. The aim of the study was to determine whether admission to hospital increases usage of beneficial HF medication and if this usage is maintained directly after discharge.

**Methods:**

The study was conducted from November 2002 until January 2004. In 77 patients hospitalised with heart failure (HF), the medication prescribed by the referring general practitioner (GP) and drug treatment directed by the hospital physicians was documented. Information regarding the post-discharge (14 d) therapy by the GP was evaluated via a telephone interview. Ejection fraction values, comorbidity and specifics regarding diagnostic or therapeutic intervention were collected by chart review.

**Results:**

When compared to the referring GPs, hospital physicians prescribed more ACE-inhibitors (58.4% vs. 76.6%; p = 0.001) and beta-blockers of proven efficacy in HF (metoprolol, bisoprolol, carvedilol; 58.4% vs. 81.8%). Aldosterone antagonists were also administered more frequently in the hospital setting compared to general practice (14.3% vs. 37.7%). The New York Heart Association classification for heart failure did not influence whether aldosterone antagonists were administered either in primary or secondary care. Fourteen days after discharge, there was no significant discontinuity in discharge medication.

**Conclusion:**

Patients suffering from HF were more likely to receive beneficial medication in hospital than prior to admission. The treatment regime then remained stable two weeks after discharge. We suggest that findings on drug continuation in different cardiovascular patients might be considered validated for patients with HF.

## Background

To date, evidence-based recommendations help doctors to manage patients with heart failure (HF) [1-5]. According to these recommendations, ACE-inhibitors (ACEI) and in case of ACEI intolerance angiotensin receptor blockers (ARB) represent the gold standard for the treatment of heart failure in all four degrees according to the New York Heart Association (NYHA) [6-9]. For beta blockers (BB), such a beneficial effect is scientifically proven for only three substances: metoprolol [10], bisoprolol [11] and carvedilol [12]. In patients with advanced HF (NYHA III-IV), aldosterone antagonists (AA) might improve pathology, endothelial function, and reduce the frequency of hospitalizations and mortality of patients [13,14].

However, the implementation of these recommendations in primary care is still problematic. Literature suggests that all beneficial drug groups mentioned above are infrequently prescribed by general practitioners (GPs) [15-20]. The persistence of out-dated treatment conceptions might be a reason for this phenomenon [21]. Uncertainty in the diagnosis of HF and a lack of communication between involved physicians can also influence whether guidelines are adhered to by GPs [15,22,23].

Prescription recommendations from hospital physicians after hospital discharge may increase the prescription rates of beneficial drugs as hospitalization seems to improve the transformation of general measures by patients with HF [24]. However, little is known about whether prescription recommendations after discharge are evidence-based and about what happens to HF medication immediately after discharge when the GP has to discuss the changes made in hospital with the patient. For the German health care system this question is notably relevant, because patients usually leave hospital just with a recommendation for further treatment and have to see their GP soon for new prescriptions. Although GPs sometimes discontinue discharge medication for their patients (e.g. acid-suppressive medications [25]), it seems to be maintained in patients with a variety of cardiovascular morbidities [26]. It is not yet known if this also applies to patients with heart failure – especially for patients with a reduced left ventricular function. The question of how and to what extent medications change when HF patients are admitted to hospital and discharged into the care of GPs has not been examined.

The aim of the study was to determine whether admission to hospital increases the usage of beneficial HF medication and if this usage is maintained directly after discharge. With a focus on patients with reduced left ventricular function (45% or less), we analysed prescription patterns prior to hospital admission, during hospitalization and 14 days after discharge.

## Methods

From November 2002 until December 2003, patients with heart failure hospitalised in the Department of Internal Medicine at the University Hospital in Göttingen were identified by the responsible doctor, clinical records and the admission form submitted by the general practitioner. The survey ended in January 2004.

### Inclusion criteria for patients

• Informed consent

• Documented diagnosis of heart failure for each NYHA class (not necessarily the reason of admission)

• Ejection fraction ≤ 45%, measured by echocardiography

• Age >18 years

• Sufficient ability to communicate in German.

### Exclusion criteria for patients

• Short stay in hospital (only one day or less)

• Inability to communicate

• Consent not given and/or inability to consent

• Severe comorbidity (e.g. cancer or terminal renal insufficiency).

### Ascertainment of medications

The medication for each patient was recorded at three points in time: directly after admission to hospital (medication prescribed by the GP), at discharge (medication specified by hospital doctors) and 2 weeks after discharge (continuation of discharge medication by GP). During hospitalization, information regarding the medication prescribed by patients' GP was recorded using a standardised questionnaire. Medication prescribed in hospital was obtained from the discharge letter. Fourteen days after discharge, each patient was contacted by telephone and the details of the current medication were recorded. We ascertained that every patient had already seen his GP after discharge from hospital. All drugs were coded according to the Anatomical Therapeutic Chemical classification system (ATC Code). CS, who collected the data during the entire study period, was trained in several training sessions. Details of the data collection were presented as well as preliminary results at several conferences with the participation of all research fellows of our department.

### Collection of clinical characteristics and statistical analysis

Ejection fraction values, comorbidity and specifics regarding diagnostic or therapeutic intervention were collected by chart review. Comorbidity was classified according to the International Classification of Diseases. The severity of disease was graded according to the NYHA classification as documented in the clinical records. Data analysis was carried out using SPSS 12.0. For differences in prescription rates, McNemar's Test was performed.

## Results

**Figure 1:**
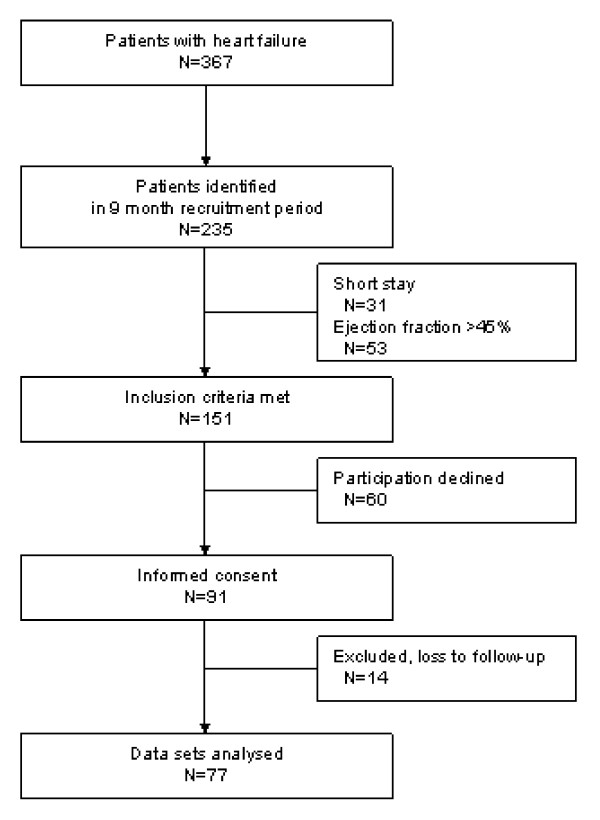
Participation in the study [26].

Of 1,785 screened patients, 235 patients with HF could be identified. Hundred-and-fifty-one patients met inclusion criteria and 91 patients were willing to participate in the study, 77 patients were analysed (figure 1): 68 men (88.3%) and 9 women (11.7%). The baseline characteristics are outlined in the table.

**Figure 2:**
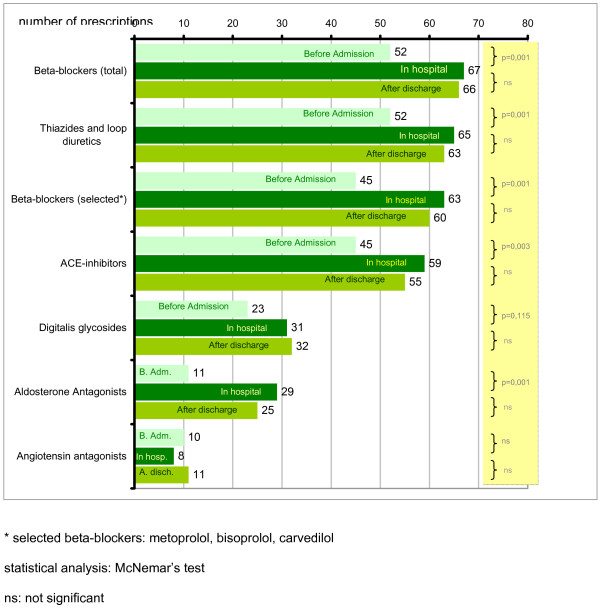
Pharmacotherapy of HF patients before admission, in hospital and 14 days after discharge.

As shown in figure 2, beneficial drug prescriptions (ACEI, BB, AA) as well as diuretics increased significantly during hospitalization. After discharge, the number of prescriptions did not decrease significantly (figure 2).

There were no significant differences in the use of ARB between pre-admission, hospitalization and post-discharge. Of the patients without ACEI medication 31.2% (10 of 32), 44.4% (8 of 18) and 50% (11 of 22) of these received ARBs before admission, in hospital and 14 days after discharge, respectively.

Hospital doctors used BB of proven efficacy in HF (metoprolol, bisoprolol, carvedilol) more frequently than GPs. This effect remained at least 14 days after discharge. During hospital treatment, metoprolol, bisoprolol or carvedilol, which improve the long-term prognosis of HF patients, represented 94.0% of all prescribed BB. Before admission, metoprolol, bisoprolol or carvedilol represented 86.5% of all BB prescriptions and after discharge this increased to 90.9%.

AA were also prescribed more often in hospital. However, patients with more severe heart failure according to the NYHA classification were not necessarily prescribed AA. In fact, fairly even numbers of patients in each of the NYHA categories, with a slightly higher prevalence in the NYHA classifications I and II, received treatment with AA: 42.1% of Class I and II in-patients obtained AA (16 of 38), while only 37.5% of patients in Groups III and IV received AA (13 of 39).

## Discussion and Conclusion

ACEI, BB of proven efficacy (metoprolol, bisoprolol, carvedilol) and AA were used significantly more often by hospital physicians than by referring GPs. Although administered more frequently to in-patients, AA were not prescribed predominantly to severely ill patients contrary to evidence-based recommendations. However, this might be due to cogent conditions (e.g. hyperkaliaemia) that could not be elaborated within our study. Fourteen days after discharge there was no significant discontinuity in discharge medication. Most of our patients received diuretics although they do not fit in the current pathophysiological model of HF. According to that model, diminishing the activation of the renine-angiotensin system and sympathical activation is thought to be essential in the treatment of patients with HF. However, possibly evidence based recommendations underestimate diuretics since all important studies on HF show the beneficial effect of ACEI, BB and AA in addition to diuretics.

The two most-striking results of our study are as follows:

1. Compared to hospital doctors, referring GPs prescribed drugs influencing long-term prognosis relatively infrequently – in spite of published recommendations.

2. AA were administered more frequently during hospital treatments but contrary to treatment guidelines, severely diseased patients did not preferentially receive this medication.

Our findings are primarily based on the analysis of prescription patterns and do not take into consideration the quality of the treatment for a particular patient. Additionally, because of the asymmetrical gender distribution in our sample, our conclusions may not be generally applicable. Whereas fourteen patients declined participation during the study (figure 1), from all remaining patients (N = 77) all three HF medications could be obtained. Validity of medication records might be reduced, because information on drug treatment two weeks after discharge was obtained by telephone. However, we are in line with some other studies that also collected medication data on basis of telephone interviews, for example the Valencia study [27] in which patients were telephoned after discharge. Moreover, several measures should contribute to the validity of the data: (1) patients were confronted with a personal survey on their medication use already in hospital so that they became familiar with these questions. (2) They were told that they could expect a phone call two weeks after discharge using similar questions. (3) Difficulties to communicate (e.g. dementia) belonged to the exclusion criteria so that we only interviewed patients with an adequate memory and sufficient intelligence. (4) Patients were told to bring all their medication to the telephone and to spell the names of the drugs.

According to current literature, the lack of adherence to discharge recommendations by primary care doctors is at least partially due to an interface problem between primary and secondary care. GPs might consider discontinuing drugs prescribed in hospital for primary care patients (e.g. acid-suppressive medications [24]). However, cardiovascular drugs are maintained by GPs in patients with a variety of cardiovascular diseases as Harder et al. suggested [25]. Our data might complement these findings on drug continuation since they also seem to apply for HF patients with decreased ejection fraction.

In our study, approximately 60% of the outpatients and almost 80% of in-patients received ACEI. BB were prescribed in about 60% of primary care patients and in 85% of hospital patients. This is in distinct contrast to a study of Rutten et al. published in 2003 [28], which found that Dutch GPs and internists prescribed ACEI and BB much less frequently (40% GPs vs. 76% internists and 9% vs. 30%, respectively) while AAs (11% vs. 76%) were prescribed at a frequency similar to that found in our study. While our study included only patients with a left ventricular function of 45% or less, Rutten et al. considered all types of HF patients and in the case of GP management, only those HF patients that were not co-treated by a cardiologist. In an earlier review [29] on treatment patterns in heart failure in nine European countries, prescription rates of ACEI and BB were noticeably lower.

Interface problems are regarded as an important factor for the continuity of evidence-based medicine [30]. Our study showed that there was no significant discontinuity in HF medication in patients discharged from hospital. Further research is needed to analyse, whether this trend also applies to the long-term prescription of beneficial drugs. With regard to the prescription rates seen in our study, there might still be potential for extended usage of established drugs in the pharmacotherapy of patients prior to or without admission to hospital.

## Competing interests

The author(s) declare that they have no competing interests.

## Authors' contributions

MS performed statistical analysis and wrote the manuscript. CS participated in the design of the study and collected the data. JK, DW and MMK participated in the design of the study and commented on the manuscript. All authors have approved the final manuscript.

**Table 1:** Baseline characteristics of the patients.

**Characteristics**	**n (%)**
Total number of patients	77
Men	68 (88.3%)
Age; mean (M) standard deviation (SD)	67.3 (± 10.8)
Duration of hospital stay in days; M (SD)	18.3 (± 14.6)
Comorbidity	
-Coronary heart disease (CHD)	40 (51.9)
-Atrial fibrillation	25 (57.1)
-Peripheral arterial disease	6 (7.8)
-Diabetes	9 (11.7)
-Chronic renal insufficiency	31 (40,2)
-Obstructive lung disease	6 (7.8)
-Gastrointestinal disorder	6 (7.8)
Reason for admission	
-Progredient HF	32 (41.6)
-Acute coronary syndrome	10 (13.0)
-Acute tachyarrhythmia	21 (27.3)
-Valvular disease	3 (3.9)
-Elective intracardiac catheter	3 (3.9)
-Other reasons	5 (6.5)
-Reason unknown	3 (3.9)
NYHA – Class	
-I	11 (14.3)
-II	27 (35.1)
-III	32 (41.6)
-IV	7 (9.1)
Ejection fraction; M (SD)	31.4 (± 8.5)

## Pre-publication history

The pre-publication history for this paper can be accessed here:


